# Considering equity and cost-effectiveness in assessing a parenting intervention to promote early childhood development in rural Vietnam

**DOI:** 10.1093/heapol/czad057

**Published:** 2023-07-26

**Authors:** Yeji Baek, Zanfina Ademi, Thach Tran, Alice Owen, Trang Nguyen, Stanley Luchters, David B Hipgrave, Sarah Hanieh, Tuan Tran, Ha Tran, Beverley-Ann Biggs, Jane Fisher

**Affiliations:** School of Public Health and Preventive Medicine, Monash University, 553 St Kilda Road, Melbourne, Victoria 3004, Australia; School of Public Health and Preventive Medicine, Monash University, 553 St Kilda Road, Melbourne, Victoria 3004, Australia; Centre for Medicine Use and Safety, Faculty of Pharmacy and Pharmaceutical Sciences, Monash University, 381 Royal Parade, Parkville, Victoria 3052, Australia; School of Public Health and Preventive Medicine, Monash University, 553 St Kilda Road, Melbourne, Victoria 3004, Australia; School of Public Health and Preventive Medicine, Monash University, 553 St Kilda Road, Melbourne, Victoria 3004, Australia; Research and Training Centre for Community Development (RTCCD), No. 6, Alley 46, Tran Kim Xuyen Street, Trung Hoa, Cau Giay District, Hanoi 10000, Vietnam; Centre for Sexual Health and HIV/AIDS Research (CeSHHAR), 4 Bath Road, Harare, Zimbabwe; Liverpool School of Tropical Medicine (LSTM), Pembroke Place, Liverpool L3 5QA, UK; UNICEF Iraq, Karadat Maryam District, Haifa Street, Baghdad 10011, Iraq; The Peter Doherty Institute for Infection and Immunity, University of Melbourne, 792 Elizabeth Street, Melbourne 3000, Australia; Research and Training Centre for Community Development (RTCCD), No. 6, Alley 46, Tran Kim Xuyen Street, Trung Hoa, Cau Giay District, Hanoi 10000, Vietnam; Research and Training Centre for Community Development (RTCCD), No. 6, Alley 46, Tran Kim Xuyen Street, Trung Hoa, Cau Giay District, Hanoi 10000, Vietnam; The Peter Doherty Institute for Infection and Immunity, University of Melbourne, 792 Elizabeth Street, Melbourne 3000, Australia; School of Public Health and Preventive Medicine, Monash University, 553 St Kilda Road, Melbourne, Victoria 3004, Australia

**Keywords:** Early childhood development, cognitive development, equity, cost-effectiveness, Vietnam

## Abstract

Considering equity in early childhood development (ECD) is important to ensure healthy development for every child. Equity-informative cost-effectiveness analysis can further guide decision makers to maximize outcomes with limited resources while promoting equity. This cost-effectiveness study aimed to examine the equity impacts of a multicomponent ECD intervention in rural Vietnam. We estimated the cost-effectiveness of the intervention with a 30-month time horizon from the service provider and household perspectives with equity considerations. Data were from a cluster-randomized controlled trial comparing the intervention with the local standard of care. The incremental cost-effectiveness ratios (ICERs) per child cognitive development score gained were estimated by household wealth quintile and maternal education level, adjusted for cluster effects and baseline characteristics such as maternal parity and age. A 3% discount rate was applied to costs, and non-parametric cluster bootstrapping was used to examine uncertainty around ICERs. Children in the intervention had higher cognitive development scores than those in the control arm across all subgroups. Based on intervention recurrent cost, the ICER per cognitive development score gained was lower in children from the poorest quintile (−US$6) compared to those from the richest quintile (US$16). Similarly, the ICER per cognitive development score gained was lower in children whose mothers had the lowest education level (−US$0.02) than those with mothers who had the highest education level (US$7). Even though our findings should be interpreted with caution due to the insufficient study power, the findings suggest that the intervention could promote equity while improving child cognitive development with greater cost-effectiveness in disadvantaged groups.

Key messages

- Ensuring child health and well-being is one of the key priority areas in Vietnam. To support mothers and children in rural Vietnam, this cost-effectiveness study aimed to examine the equity impacts of an early childhood development intervention.
- With a 30-month time horizon, the intervention was more cost-effective in children in the poorest two quintiles than those in the higher wealth quintiles and more cost-effective in children with mothers who had less educated mothers than those with more educated mothers.
- Even though our findings should be interpreted with caution due to the insufficient study power, the findings suggest that the intervention could promote equity while improving child cognitive development with greater cost-effectiveness in disadvantaged groups.

## Introduction

The world has achieved reductions in child mortality with efforts to combat poverty and hunger. Globally, the under-5 mortality rate decreased by 59% from 93.0 deaths per 1000 live births in 1990 to 37.7 in 2019 (Sharrow *et al.*, 2022), and the coverages of reproductive, maternal, newborn, and child health interventions had improved (Countdown to 2030 Collaboration, 2018). Accordingly, the global agenda has shifted to an increased focus on promoting health and well-being. Despite the progress, disparities persist with more deaths and greater challenges to child health and well-being in disadvantaged groups. A study based on national surveys from 94 low- and middle-income countries showed that fewer children in rural areas or the lowest household wealth quintile were exposed to home stimulation such as singing and playing, and fewer attended early care and education compared to those in urban areas or in the richest wealth quintile (Lu *et al.*, 2020).

The analyses of cohort data found that linear growth during the first 2 years of life was a strong predictor of educational attainment and adult intelligence quotients (Black *et al.*, 2022), which highlights the importance of ensuring child development. Two Lancet Series in 2016 and 2022 on child development and the Nurturing Care Framework emphasized a holistic approach across health, education, and social systems to ensure children’s good health and nutrition and protect them from threats (Britto *et al.*, 2017; World Health Organization *et al.*, 2018; Black *et al.*, 2022). Among early childhood development (ECD) trials in low- and middle-income countries (Muhoozi *et al.*, 2018; Rockers *et al.*, 2018; Galasso *et al.*, 2019; Abimpaye *et al.*, 2020; Grantham-McGregor *et al.*, 2020; Mehrin *et al.*, 2022), only one study examined the equity impacts of a parenting education programme (Abimpaye *et al.*, 2020). The study, in Rwanda, found that children with more educated mothers or from wealthier families were more likely to meet developmental milestones than those with less educated mothers or from poorer families (Abimpaye *et al.*, 2020). To our knowledge, no studies have examined the cost-effectiveness of ECD interventions with equity considerations in low- and middle-income countries. There are missed opportunities to identify who benefits more from interventions or who is left behind and whether interventions reduce or increase inequalities. Understanding the equity impacts of programmes provides meaningful information to refine strategies to reach the most disadvantaged group and achieve equity. Equity-informative cost-effectiveness analysis can guide decision-makers to maximize outcomes with limited resources while promoting equity. Furthermore, it contributes to the key principles of the 2030 Agenda for Sustainable Development, ‘leave no one behind’ and ‘reach the furthest behind first’, to end discrimination and reduce the inequalities that undermine the potential of individuals and of humanity as a whole (United Nations Sustainable Development Group, 2022).

Ensuring child health and well-being is one of the key priority areas in Vietnam. However, the World Bank Group Human Capital Index 2020 estimated that children born in Vietnam today would be 69% as productive when they grow up as they could be with complete education and full health (World Bank, 2020). To support mothers and children in rural Vietnam, a multicomponent ECD intervention was conducted, and it was found to benefit child cognitive, language, and motor development and to be cost-effective (Baek *et al.*, 2023; Fisher *et al.*, 2023). Building on it, this study aimed to examine the equity impacts of the intervention by estimating the distribution of costs and effects across the socioeconomic groups.

## Methods

### Study setting

This trial was conducted in Ha Nam, a rural Red River delta province in northern Vietnam from 2018 to 2020. According to the census in 2021, the population in the province was 875 200, the under-5 mortality rate per 1000 live births was 18 and the average age of first marriage was 26 years (General Statistics Office, 2022). The monthly average income per capita was 4372 thousand Vietnamese dong (VND) (General Statistics Office, 2022), which is around US$190.

### Study design and intervention

This study is based on a cluster-randomized controlled trial comparing a multicomponent ECD intervention, ‘Learning Clubs’, with the usual standard of maternal and child healthcare in rural Vietnam (Fisher *et al.*, 2018; 2023). The study protocols have been published elsewhere (Fisher *et al.*, 2018; Nguyen *et al.*, 2019). Study findings showed that the intervention improved child cognitive, language, and motor development, and it was cost-effective with a 30-month time horizon (18 months of intervention and a 12-month follow-up period) (Baek *et al.*, 2023; Fisher *et al.*, 2023). In brief, the intervention addressed maternal nutrition and mental health, parenting capabilities, infant health and development, and gender norms through eight group sessions during pregnancy, one home visit after childbirth, and 11 group sessions during the first postpartum year. All women aged at least 18 years, who were pregnant and with gestation less than 20 weeks were eligible to participate. Potential participants were informed at the commune health centres or through local loudspeaker announcements, and they were invited for recruitment upon their consent. Mothers in the intervention arm attended sessions from mid-pregnancy to when their children were 1 year old. Other caregivers including fathers and grandparents also joined the sessions when feasible. In addition to the sessions, mothers were able to access their usual maternal and child healthcare from commune health services (pregnancy checks, birth in a medical facility, and national growth monitoring and immunization programmes). Mothers in the control arm received the usual standard of maternal and child healthcare alone.

As outlined in previous studies, the primary outcome was child cognitive development composite score at 2 years of age assessed by the Bayley Scale of Infant and Toddler Development Third Edition (Bayley-III). The number of clusters and sample size were determined to detect a difference in the proportion of children scoring <1 SD on the Bayley-III of 15% in the control arm and 8% in the intervention arm (with 80% statistical power and a significance level of 0.05; intracluster correlation coefficient = 0.03) (Fisher *et al.*, 2018; 2023). A total of 1008 pregnant women from 84 communes (504 women from 42 communes in each trial arm) were needed (Fisher *et al.*, 2018; 2023). An independent statistician selected 84 communes randomly among 112 communes in the Province and allocated 42 communes randomly to each trial arm using random numbers generated in Stata V.14.0 (Fisher *et al.*, 2018; 2023). The trial was not powered to detect subgroup effects because an equity analysis was not planned beforehand.

This study followed Consolidated Standards of Reporting Trials-Equity guidelines 2017 (Welch *et al.*, 2017) and Consolidated Health Economic Evaluation Reporting Standards 2022 (Husereau *et al.*, 2013).

### Outcome and cost measures

Outcome and cost measures were reported in Learning Clubs effects and cost-effectiveness studies (Baek *et al.*, 2023; Fisher *et al.*, 2023). The primary outcome of the trial was child cognitive development at the age of 2 years assessed by the Bayley-III. The cognitive sub-scale assesses sensorimotor manipulation and exploration, early memory and problem-solving skills and concept formation (Albers and Grieve 2007). The scores were converted to composite scores adjusted for child age and sex with a mean of 100 and a SD of 15 (ranging from 40 to 160) in line with previous studies (Baek *et al.*, 2023; Fisher *et al.*, 2023) and its guidelines (Bayley, 2006).

Costs were collected from the service provider and household perspectives including intervention costs, mother’s time to participate in the intervention, and out-of-pocket healthcare costs, as outlined in the cost-effectiveness study (Baek *et al.*, 2023). Intervention cost data were taken from the cost-effectiveness study, which included start-up cost (package development, materials and supplies, workshops and training) and recurrent cost (personnel, Learning Clubs sessions, supervision/management, and household participation) (Baek *et al.*, 2023). As for out-of-pocket healthcare costs, inpatient and outpatient costs such as medication, medical examination, and hospitalization costs for maternal healthcare during pregnancy and child healthcare from birth to 12 months were collected through structured interviews (Baek *et al.*, 2023). Costs were collected in VND in 2018–19 and converted to US dollars (US$1 = 23,050.24 VND) (International Monetary Fund & International Financial Statistics).

### Equity measures

We conducted subgroup analyses based on household wealth and mother’s education at baseline to examine how costs and effects are distributed by socioeconomic groups. Household Wealth Index was calculated according to the World Bank method (O’Donnell *et al.*, 2008) considering household characteristics (drinking water source, cooking fuels, type of latrine, number of household members per room, and materials of walls, floor, and roof), and assets (vehicles, furniture, land, and livestock). Participants were then divided into quintiles with the bottom 20% categorized as the poorest (Quintile 1) and the top 20% categorized as the richest (Quintile 5). Mother’s education level was categorized as ‘Secondary (up to Year 9) or lower’, ‘High school (up to Year 12)’, and ‘College/university degree and higher’.

### Analysis

We followed the similar methods as the cost-effectiveness study of the ‘Learning Clubs’ cluster-randomized trial (Baek *et al.*, 2023). The costs and effects by household wealth and mother’s education level were examined to measure the equity impacts of the intervention. The differences in costs and effects between the intervention and control arms were estimated for each subgroup using least squares means based on generalized linear mixed models adjusting for cluster effects and baseline characteristics. The differences in costs and effects by household wealth quintile were adjusted for the number of household members, parity, mother’s age, mother’s occupation, father’s age, father’s education, father’s occupation, and mother’s education. Similarly, the differences in costs and effects by mother’s education level were adjusted for the number of household members, parity, mother’s age, mother’s occupation, father’s age, father’s education, father’s occupation, and household wealth. The costs and effects are presented as mean and 95% confidence interval (CI). Tests of interactions between socioeconomic groups and trial arms on the effects were performed.

Multiple imputations were used to handle missing data on out-of-pocket healthcare costs based on the log multiple imputation predictive mean matching algorithm as reported in the cost-effectiveness study (Baek *et al.*, 2023). A 3% discount rate was applied to costs that occurred after first year following the WHO’s methods (Bertram *et al.*, 2021) and previous cost-effectiveness study (Baek *et al.*, 2023).

The incremental cost-effectiveness ratios (ICERs) were estimated by dividing the mean difference in costs by the mean difference in effects for each subgroup based on household wealth and mother’s education level. In addition to estimating ICERs based on intervention cost and out-of-pocket healthcare cost adjusting for cluster effects and baseline characteristics, we also estimated ICERs under different scenarios that include intervention cost alone without out-of-pocket healthcare costs or results adjusting for cluster effects. We used non-parametric cluster bootstrapping by randomly resampling clusters with replacement and presented mean and 95% CI of ICERs from 1000 bootstrap replications by subgroups. The bootstrap estimates were plotted on the cost-effectiveness plane and used to estimate the probability that the intervention was cost-saving or cost-effective. Since there was no national cost-effectiveness threshold per child cognitive score gained, we used alternative threshold of US$56, which is 2% of Vietnam’s gross domestic product (GDP) (World Bank Group) based on the G20’s investment benchmark for ECD (Richter *et al.*, 2018).

All analyses used the SAS 9.4 software and Microsoft Excel Office 2019.

## Results

### Baseline characteristics

The two arms (622 infants in intervention; 546 infants in control) had comparable household wealth status and mother’s education levels (Table 1). Maternal parity was slightly lower in the richest quintile compared to the other wealth quintiles in both arms. Similarly, maternal parity was lowest among mothers with college/university degree and higher compared to those in less educated mothers. Other baseline characteristics including mother’s occupation, father’s age, father’s education, and father’s occupation are presented in Supplementary Table 1.

**Table 1.** Baseline characteristics by household wealth quintile and mother’s education level

	Intervention (*n* = 622)		Control (*n* = 546)	
Household wealth quintile	*N*	%	*N*	%
Quintile 1 (poorest)	119	19.1	113	20.7
Quintile 2	123	19.8	113	20.7
Quintile 3	113	18.2	125	22.9
Quintile 4	136	21.9	96	17.6
Quintile 5 (richest)	131	21.1	99	18.1
**Mother’s education**	***N***	**%**	***N***	**%**
Secondary (up to Year 9) or lower	239	38.4	210	38.5
High school (up to Year 12)	189	30.4	164	30.0
College/university degree and higher	194	31.2	172	31.5
**Parity**	**Mean**	**SD**	**Mean**	**SD**
**Household wealth quintile**				
Quintile 1 (poorest)	1.5	0.8	1.5	0.6
Quintile 2	1.5	0.7	1.6	0.7
Quintile 3	1.5	0.7	1.4	0.7
Quintile 4	1.5	0.6	1.3	0.6
Quintile 5 (richest)	1.3	0.6	1.3	0.7
**Mother’s education**				
Secondary (up to Year 9) or lower	1.7	0.8	1.6	0.7
High school (up to Year 12)	1.4	0.6	1.3	0.6
College/university degree and higher	1.2	0.5	1.2	0.6
**Mother’s age in years**	**Mean**	**SD**	**Mean**	**SD**
**Household wealth quintile**				
Quintile 1 (poorest)	27.7	5.5	27.9	5.8
Quintile 2	26.5	5.4	27.1	5.6
Quintile 3	27.5	5.3	26.6	5.2
Quintile 4	27.7	5.2	27.1	5.5
Quintile 5 (richest)	28.2	5.3	26.9	5.0
**Mother’s education**				
Secondary (up to Year 9) or lower	28.8	6.2	28.3	6.7
High school (up to Year 12)	25.9	5.0	25.3	4.7
College/university degree and higher	27.6	4.0	27.4	3.7

### Equity impact on cost-effectiveness

**Figure 1.**
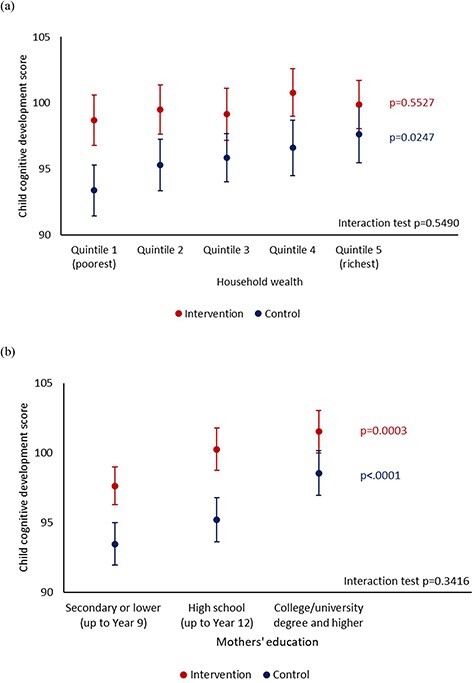
Child cognitive score by (a) household wealth quintile and (b) mother’s education level. *P* values correspond to comparison across the socioeconomic groups. Interaction test between socioeconomic groups and trial arms on the outcome.

We assessed child cognitive development score across socioeconomic groups by trial arms (Figure 1). Overall, child cognitive development score was higher in the intervention arm than the control arm in all subgroups. The score in the intervention arm was closer to or higher than the normative mean of 100. In the control arm, cognitive development score was different by household wealth quintile (*P* = 0.0247), with the lowest score in the poorest quintile and highest score in the richest quintile. However, there was no significant difference by household wealth quintile in the intervention arm. Child cognitive development score was different by mother’s education level in both intervention (*P* = 0.0003) and control arms (*P* < 0.0001). Children with mothers who had up to secondary (nine years) education had the lowest cognitive score compared to those with mothers who had high school or college/university degree and higher education. We did not find an interaction effect between trial arms and subgroups on the child cognitive development score.

The difference in cognitive development score between the intervention and control arms was statistically significant in children from the poorest quintile but not in those from richest quintile (Table 2). When adjusting for cluster effects and baseline characteristics, the mean difference in score was 6.8 (95% CI 3.8 to 9.9) in the poorest and 3.1 (95% CI −0.9 to 7.2) in the richest quintile, indicating greater benefits in the poorest quintile. The mean difference in score was 5.3 (95% CI 3.3 to 7.3) for children with mothers who had secondary or lower education and 5.1 (95% CI 2.1 to 8.1) for those with mothers who had college/university degree and higher.

There was a wide range of 95% CI for out-of-pocket healthcare costs in all subgroups with no significant difference between the trial arms (Table 2). As a universal intervention, the intervention cost per child was the same across subgroups in the intervention arm at US$273 for start-up and recurrent cost and at US$58 for recurrent cost (Baek *et al.*, 2023).

**Table 2.** Cost-effectiveness to improve child cognitive development by household wealth quintile and mother’s education level (base-case)

	Adjusted for cluster effects						Adjusted for cluster effects and baseline characteristics^b^	
	Intervention		Control		Difference		Difference	
	Mean	(95% CI)	Mean	(95% CI)	Mean	(95% CI)	Mean	(95% CI)
Child cognitive score by household wealth								
Quintile 1 (poorest)	98.6	(96.8 to 100.3)	93.2	(91.5 to 95.0)	5.3	(2.8 to 7.8)	6.8	(3.8 to 9.9)
Quintile 2	99.4	(97.8 to 100.9)	95.4	(93.8 to 97.0)	4.0	(1.8 to 6.2)	6.7	(4.0 to 9.3)
Quintile 3	99.3	(97.1 to 101.5)	95.9	(93.9 to 97.9)	3.4	(0.5 to 6.4)	5.3	(1.6 to 9.0)
Quintile 4	100.8	(98.8 to 102.8)	96.6	(94.4 to 98.9)	4.2	(1.2 to 7.2)	4.1	(0.9 to 7.3)
Quintile 5 (richest)	99.9	(98.0 to 101.9)	97.3	(95.0 to 99.6)	2.7	(−0.3 to 5.7)	3.1	(−0.9 to 7.2)
**Child cognitive score by mother’s education**								
Secondary or lower	97.6	(96.3 to 98.8)	93.5	(92.1 to 94.8)	4.1	(2.3 to 5.9)	5.3	(3.3 to 7.3)
High school	100.3	(98.7 to 101.8)	95.2	(93.5 to 96.8)	5.1	(2.9 to 7.4)	6.0	(2.9 to 9.1)
College/university degree and higher	101.5	(99.8 to 103.3)	98.6	(96.7 to 100.4)	3.0	(0.4 to 5.5)	5.1	(2.1 to 8.1)
**Out-of-pocket healthcare cost by household wealth**								
Quintile 1 (poorest)	$131	(6 to 257)	$158	(33 to 283)	−$27	(−204 to 151)	−$96	(−252 to 62)
Quintile 2	$123	(14 to 231)	$184	(68 to 301)	−$62	(−221 to 98)	−$116	(−347 to 115)
Quintile 3	$161	(71 to 252)	$126	(37 to 215)	$35	(−92 to 162)	−$24	(−173 to 126)
Quintile 4	$169	(113 to 224)	$107	(43 to 170)	$62	(−23 to 146)	$24	(−90 to 138)
Quintile 5 (richest)	$171	(43 to 300)	$195	(47 to 344)	−$24	(−219 to 172)	−$8	(−219 to 203)
**Out-of-pocket healthcare cost by mother’s education**								
Secondary or lower	$114	(26 to 201)	$215	(121 to 309)	−$101	(−230 to 28)	−$58	(−188 to 72)
High school	$165	(98 to 232)	$125	(56 to 193)	$40	(−56 to 136)	$56	(−96 to 209)
College/university degree and higher	$185	(110 to 259)	$109	(25 to 193)	$76	(−37 to 189)	−$20	(−119 to 79)
**Intervention cost per child** ^a^								
Start-up and recurrent cost	$273				$273		$273	
Recurrent cost	$58				$58		$58	
	**Based on start-up and recurrent cost**				**Based on recurrent cost**			
**ICER per cognitive score gained**	**Adjusted for cluster effects**		**Adjusted for cluster effects and baseline characteristics**		**Adjusted for cluster effects**		**Adjusted for cluster effects and baseline characteristics**	
**Household wealth quintile**								
**Intervention and out-of-pocket healthcare cost**								
Quintile 1 (poorest)	$46		$26		$6		−$6	
Quintile 2	$53		$24		−$1		−$9	
Quintile 3	$89		$47		$27		$7	
Quintile 4	$80		$73		$29		$20	
Quintile 5 (richest)	$94		$84		$13		$16	
**Intervention cost only (without out-of-pocket healthcare cost)**								
Quintile 1 (poorest)	$51		$40		$11		$9	
Quintile 2	$68		$41		$15		$9	
Quintile 3	$79		$51		$17		$11	
Quintile 4	$65		$67		$14		$14	
Quintile 5 (richest)	$103		$87		$22		$19	
**Mother’s education**								
**Intervention and out-of-pocket healthcare cost**								
Secondary or lower	$42		$41		−$10		−$0.02	
High school	$61		$55		$19		$19	
College/university degree and higher	$118		$50		$45		$7	
**Intervention cost only (without out-of-pocket healthcare cost)**								
Secondary or lower	$67		$52		$14		$11	
High school	$53		$45		$11		$10	
College/university degree and higher	$92		$54		$20		$11	

Costs are in US$ 2019.

a Intervention cost data from the trial’s main cost-effectiveness study (Baek *et al.*, 2023).

b Outcome and cost by household wealth quintile are adjusted for the number of household members, parity, mother’s age, mother’s occupation, father’s age, father’s education, father’s occupation and mother’s education. Outcome and cost by mother’s education are adjusted for the number of household members, parity, mother’s age, mother’s occupation, father’s age, father’s education, father’s occupation and household wealth.

The base-case ICER per cognitive development score gained showed that the intervention was more cost-effective in children from two poorest quintiles than richer quintiles (Table 2). Based on mother’s education level, the intervention was more cost-effective in children with mothers who had secondary or lower education compared to those with mothers who had high school or college and higher education. Negative ICERs indicated that the intervention was cost-saving in children from two poorest quintiles and those with mothers who had secondary or lower education based on intervention recurrent cost.

**Figure 2.**
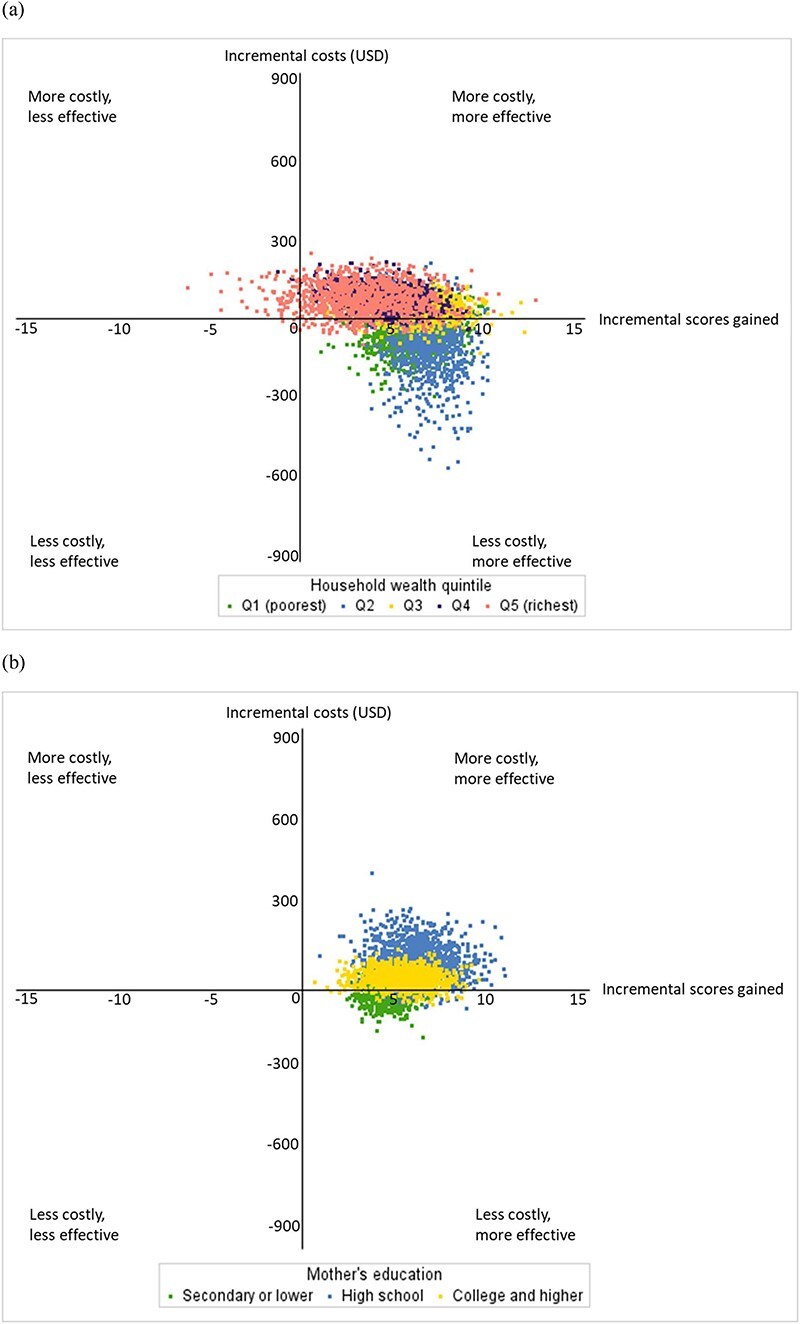
Cost-effectiveness plane of 1000 bootstrap iterations by (a) household wealth quintile and (b) mother’s education level based on intervention recurrent cost and out-of-pocket healthcare cost. Costs are in US dollars (USD) 2019.

The mean ICER of bootstrap samples ranged from −US$13 (95% CI −62 to 21) in Quintile 2 to US$25 in Quintile 4 (95% CI −2 to 87) based on intervention recurrent cost when adjusting for cluster effects and baseline characteristics (Table 3 and Figure 2). Based on mother’s education level, the mean ICER of bootstrap samples ranged from −US$0.1 (95% CI −21 to 18) in children whose mothers had lowest education level to US$20 (95% CI −2 to 55) in children whose mothers had highest education level. Among 1000 bootstrapped estimates based on intervention recurrent cost, over 97% of estimates were either cost-saving or cost-effective in all subgroups except those in the two richest quintiles. Nearly 70% of estimates were cost-saving, and 30% of estimates were cost-effective in the two poorest quintiles.

**Table 3.** ICERs per child cognitive development score gained by household wealth quintile and mother’s education level (1000 times bootstrapping)

	Based on start-up and recurrent cost				Based on recurrent cost					
	Adjusted for cluster effects		Adjusted for cluster effects and baseline characteristics^b^		Adjusted for cluster effects		Adjusted for cluster effects and baseline characteristics^a^		Cost-saving^b^	Cost-effective^b^
	Mean	95%CI	Mean	95%CI	Mean	95%CI	Mean	95%CI	%	%
**Household wealth quintile**										
**Intervention and out-of-pocket healthcare cost**										
Quintile 1 (poorest)	$73	(19 to 156)	$29	(1 to 65)	$28	(−23 to 89)	−$9	(−42 to 16)	67.5	32.5
Quintile 2	$49	(13 to 108)	$21	(−29 to 60)	−$4	(−33 to 29)	−$13	(−62 to 21)	69.7	30.3
Quintile 3	$98	(42 to 257)	$47	(23 to 98)	$29	(2 to 86)	$7	(−9 to 27)	18.6	81.3
Quintile 4	$88	(40 to 191)	$83	(33 to 246)	$31	(8 to 74)	$25	(−2 to 87)	3.0	89.3
Quintile 5 (richest)	$80	(−542 to 692)	$69	(−544 to 847)	$8	(−175 to 226)	$4	(−142 to 223)	10.4	65.5
**Intervention cost only (without out-of-pocket healthcare cost)**										
Quintile 1 (poorest)	$56	(36 to 96)	$49	(31 to 81)	$12	(8 to 21)	$10	(7 to 17)	0	100
Quintile 2	$67	(45 to 114)	$42	(30 to 62)	$14	(10 to 24)	$9	(6 to 13)	0	100
Quintile 3	$87	(43 to 218)	$50	(30 to 106)	$18	(9 to 46)	$11	(6 to 23)	0	99.9
Quintile 4	$73	(38 to 146)	$74	(36 to 200)	$16	(8 to 31)	$16	(8 to 43)	0	98.2
Quintile 5 (richest)	$93	(−477 to 630)	$83	(−481 to 796)	$20	(−102 to 134)	$18	(−102 to 170)	0	84.6
**Mother’s education**										
**Intervention and out-of-pocket healthcare cost**										
Secondary or lower	$44	(10 to 88)	$47	(26 to 76)	−$13	(−44 to 17)	−$0.1	(−21 to 18)	49.0	51.0
High school	$61	(38 to 95)	$56	(27 to 111)	$18	(4 to 35)	$20	(−2 to 55)	3.9	93.7
College/university degree and higher	$127	(60 to 397)	$52	(29 to 100)	$53	(15 to 179)	$9	(−4 to 26)	10.7	89.2
**Intervention cost only (without out-of-pocket healthcare cost)**										
Secondary or lower	$73	(52 to 106)	$60	(43 to 89)	$15	(11 to 23)	$13	(9 to 19)	0	100
High school	$54	(38 to 79)	$46	(30 to 78)	$12	(8 to 17)	$10	(6 to 17)	0	99.9
College/university degree and higher	$95	(51 to 281)	$55	(35 to 104)	$20	(11 to 60)	$12	(7 to 22)	0	99.9

Costs are in US$ 2019. ICER = Incremental cost-effectiveness ratios.

a Outcome and cost by household wealth quintile are adjusted for the number of household members, parity, mother’s age, mother’s occupation, father’s age, father’s education, father’s occupation, and mother’s education. Outcome and cost by mother’s education are adjusted for the number of household members, parity, mother’s age, mother’s occupation, father’s age, father’s education, father’s occupation, and household wealth.

b The probability that the intervention was cost-saving or cost-effective was estimated based on 1000 bootstrapped estimates. The estimates in the north-east quadrant on the cost-effectiveness plane (Figure 2) indicate that the intervention is cost-effective, and the estimates in the south-east quadrant indicate that the intervention is cost-saving. The cost-effectiveness threshold was 2% of Vietnams’ GDP based on the G20’s investment benchmark for ECD (Richter *et al.*, 2018).

## Discussion

This study assessed the impacts on equity of the multicomponent ECD ‘Learning Clubs’ intervention, which has previously been found to be an effective intervention to improve child development (Fisher *et al.*, 2023). Our findings suggest that the intervention is likely to promote equity while improving child cognitive development in rural Vietnam. Two-year-old children in the intervention arm demonstrated better cognitive development than those in the control arm, and there was a greater benefit to the children in most disadvantaged households. With a 30-month time horizon, the intervention was more cost-effective in children in the poorest two quintiles than those in the higher wealth quintiles and more cost-effective in children whose mothers were less educated than in children whose mothers were more highly educated.

Household wealth and maternal education are well-known social determinants of children’s health. Multi-country studies have found that around half of the total deaths in children aged under-5 were in those in the poorest two quintiles (Chao *et al.*, 2018). Another study estimated a 31% reduction in mortality for children born to mothers with secondary education compared with those born to mothers with no education (Balaj *et al.*, 2021). These factors were also associated with child cognitive development in our study. This is similar to a previous study from Rwanda, which showed that maternal education and family wealth were positively related to child development, mother–child learning and playing activities and discipline behaviours (Abimpaye *et al.*, 2020). A study from Vietnam that analysed the population-based datasets found socioeconomic, regional and urban-rural inequalities in reproductive, maternal, newborn, and child health intervention coverages (Nguyen *et al.*, 2021). Our findings support the existing evidence that children from higher socioeconomic backgrounds are more likely to meet their development potential. In addition to household wealth and maternal education, a review study from Vietnam identified informal payments for healthcare, discrimination and negative attitudes from health staff towards women and ethnic minorities as determinants of inequity in maternal and child health (Målqvist *et al.*, 2012). Further research to understand the pathways of inequities in health and to suggest interventions for policy action to reach disadvantaged populations was recommended (Målqvist *et al.*, 2012). Development disparities established in early life can lead to lifetime differences with negative implications for adult functioning, next generation and the well-being of societies (Walker *et al.*, 2011), and thus, equity consideration in planning, implementing, and evaluating interventions is important.

Research evidence in equity-informative cost-effectiveness of ECD intervention is scarce. To our knowledge, no existing studies have examined the distributional cost-effectiveness of multicomponent ECD interventions in low- and middle-income countries. A scoping review on equity in economic evaluations of ECD interventions in low- and middle-income countries identified that most studies solely focused on health, and no study measured child cognitive, language, motor or social and emotional development (Baek *et al.*, 2023). In this study, we showed that our intervention was more cost-effective in children in the poorest two quintiles or children with less educated mothers compared with those from higher socioeconomic backgrounds. Economic evidence is crucial for decision-makers to maximize benefits with limited resources. Considering equity can provide further insights into the differential budget impacts and child development outcomes by social groups to ensure fair opportunities for every child.

Better value for money of interventions for children from low socioeconomic backgrounds does not mean that ECD policies and programmes should only target the poorest children. Marmot and colleagues argued that focusing solely on the most disadvantaged may stigmatize them and weaken social cohesion across the population (Marmot *et al.*, 2010; 2020). Furthermore, they argued that it will not reduce inequalities sufficiently because health inequalities are not confined to the poor, but rather health and development follow a social gradient (Marmot *et al.*, 2010; 2020). They proposed ‘proportionate universalism’, which ensures universal policies and interventions, but with an intensity that is proportionate to the level of disadvantage (Marmot *et al.*, 2010; 2020). Considering that our study participants from rural areas are likely to be less advantaged than those from urban areas, everyone in rural areas would benefit from interventions like this. To improve equity, providing additional support such as home visiting, nutritional supplements, and education or cash transfer to the most disadvantaged group may be considered. However, some challenges still remain such as which indicator and threshold to apply when identifying the level of disadvantage, how to demonstrate effective reduction of social gradient of health (Francis-Oliviero *et al.*, 2020) and how to improve cost-effectiveness.

Our findings should be interpreted with some caution considering study limitations. First, the study was not powered to detect subgroup differences as this equity analysis was not planned beforehand. A descriptive assessment study noted that many studies have been underpowered for subgroup analyses because sample size calculations are usually based on comparison between trial arms rather than on differential effects within subgroups (Petkovic *et al.*, 2020). However, despite insufficient power, findings could be used for hypothesis generation and meta-analyses or other studies where greater power could be achieved (Petkovic *et al.*, 2020). Second, due to insufficient study power, this study only examined the primary outcome and cognitive development, even though secondary outcomes including child language, motor, and socio-emotional development were measured in the trial. The intervention was found to be effective in improving child cognitive, language, and motor development (Fisher *et al.*, 2023). We acknowledge that considering all four domains of child development would provide a more comprehensive understanding as a whole. In addition, our subgroup analyses are based on household wealth and maternal education, but there are multiple factors that could affect equity. The PROGRESS-Plus equity framework refers to place of residence, race/ethnicity/culture/language, occupation, gender/sex, religion, education, socio-economic status and social capital plus personal characteristics associated with discrimination such as age and disability, features of relationships such as smoking parents and time-dependent relationships such as leaving the hospital (O’Neill *et al.*, 2014). Understanding equity requires comprehensive and context-based data. Lastly, cost-effectiveness across different subgroups was explored for over a short time horizon. The long-term equity impacts of the intervention are unknown.

## Conclusion

The ‘Learning Clubs’ intervention is likely to be more cost-effective in children from low socioeconomic backgrounds than those from high socioeconomic backgrounds. Even though our findings should be interpreted with caution due to the insufficient study power, the findings suggest that the intervention could promote equity with greater cost-effectiveness in disadvantaged groups.

## Data Availability

The data will be shared on reasonable request to the corresponding author considering privacy concerns.

## Abbreviations

Bayley-III = Bayley Scales of Infant and Toddler Development Third Edition

CI = Confidence Interval

ECD = Early Childhood Development

ICER = Incremental cost-effectiveness ratios

SD = Standard deviation

VND = Vietnamese dong

## Reflexivity statement

The investigators include nine women and three men. Four of them are Vietnamese, five of them are Australian, one is Australian and Kosovar, one is South Korean and one is Dutch. Three authors are based in Vietnam, one in Iraq, one in Zimbabwe and seven in Australia. The authors are at different levels of seniority from PhD Candidate to Professor in the areas of global health, health economics, and epidemiology.
